# Symposium “International Medical Students – Support Programs in Practical Application”: Networking, best-practice examples and local representation

**DOI:** 10.3205/zma001199

**Published:** 2018-11-30

**Authors:** Henrike Schulze, Danmei Zhang, Ricardo Patricio Pérez Anderson, Obada Alhalabi, Daniel Huhn, Timo Astfalk

**Affiliations:** 1 Medizinische Hochschule Hannover, Hannover, Germany; 2 Project International Medical Students at the German Medical Students’ Association (bvmd), Berlin, Germany; 3 Ludwig-Maximilians-University Munich, Munich, Germany; 4 University of Heidelberg, Heidelberg, Germany; 5 University of Heidelberg, Department of General Internal and Psychosomatic Medicine, Heidelberg, Germany; 6 University Medical Center Rostock, Rostock, Germany

**Keywords:** International medical students, cultural diversity, tutorials, language teaching, social learning, integration, curriculum development, medical education

## Abstract

The new project "International Medical Students" within the German Medical Students' Association (bvmd) aims at connecting local support programs for international medical students as well as the representation of their interests within the bvmd. Within the frame of this project, the first symposium "International Medical Students – Support Programs in Practical Application" took place from the 12^th^ to the 14^th^ of May 2017 at the Hannover Medical School (MHH). Through partaking in different workshops, 31 participants discussed the framework conditions of local work (i.e. curricula, tutorials, social offers, cooperation between faculty members and student body, legal aspects), common problems (i.e. addressing the target group, funding of support programs) and possible solutions (i.e. targeted advertisement, application for public funds). This report constitutes a summary of the results of these discussions. The feedback from the participants on the need for such a regular exchange and the format of the symposium was positive. However, there were requests for further thematic specification. Based on this feedback the next symposium is planned for 2018.

## Zusammenfassung

Das Projekt „Internationale Medizinstudierende“ ist ein junges Projekt innerhalb der Bundesvertretung der Medizinstudierenden in Deutschland e.V. (bvmd). Es zielt auf die Vernetzung lokaler Supportprogramme für internationale Medizinstudierende sowie deren Interessenvertretung innerhalb der medizinischen Studierendenschaft ab. Im Rahmen dieser Arbeit fand vom 12. bis 14. Mai 2017 das erste Symposium „Internationale Medizinstudierende – Supportprogramme in der Praxis“ an der Medizinischen Hochschule Hannover (MHH) statt. Die 31 Teilnehmenden diskutierten in elf Workshops über die Rahmenbedingungen der lokalen Arbeit (Curricula, Tutorien, soziale Angebote, Kooperation zwischen Fakultät und Studierendenschaft, juristische Aspekte), häufige Problemlagen (Ansprache der Zielgruppe, Finanzierung von Supportprogrammen) und mögliche Lösungen (gezielte Werbung, Beantragung von öffentlicher Mitteln). Der vorliegende Bericht stellt eine Zusammenfassung der Ergebnisse dieser Diskussionen dar. Die Rückmeldungen der Teilnehmenden zum Bedarf solch eines regelmäßigen Austausches sowie dem Format des Symposiums waren positiv, allerdings wurde eine thematische Konkretisierung gewünscht. Vor diesem Hintergrund ist eine Wiederholung des Formats für das Jahr 2018 geplant.

## 1. Introduction

As the increasing numbers of enrolled foreign students show, Germany is a desirable location for prospective students worldwide [1]. Despite strict quota regulations for students from countries outside the European Union [2], this development has also been noticed at German medical schools. Although the increasing numbers of international students are wished-for by the federal government, the rectors' conference and the universities [3], [4], they also entail local challenges. These include, in particular, lower examination performances [5], [6], longer durations of study [5], [7], reduced personal well-being [8], [9] and integration difficulties [8] among international students. The causes of these problems are manifold and often sought in language barriers [10], [11], cultural differences [12], [13] and learning socialization [14], [15]. They are, however, interconnected in a complex way. Communication barriers, for example, may arise due to both poor language skills and cultural misunderstandings. 

In order to address these diverse problems, local support programs have been developed at medical schools in recent years [16], [17], [18]. 

Nevertheless, the German and international literature on support programs for international medical students is – in contrast to research on the academic problems of international medical students – limited. Although the first international reports on support programs at foreign medical faculties are much older than the ones at German universities, similar elements can be identified. In particular, mentoring programs and counseling services appear to play a key role abroad [19], [20], [21], [22].

In Germany, the offers range from general counseling services to tutorial programs and multi-faceted support offers [12], [23]. While a call for research by Chenot et al. in 2007 [24] led to a thorough evaluation of the academic problems of international students, the evaluation of local support programs still stands at its beginning. The same applies to national and international networking as well as mutual exchange among such support programs. Consequently, surveys among German study deaneries and the attendees of the 2013 Meeting of the Society for Medical Education [12], [23] found that many faculty members are still unaware of the offers available to international students at their universities.

The new project “International Medical Students” of the German Medical Students' Association (bvmd) [https://www.bvmd.de/unsere-arbeit/projekte/internationale-medizinstudierende/] has been tackling this problem since May 2016 and is offering an exchange forum for committed students who support international medical students at their universities. With this purpose, the first symposium “International Medical Students – Support Programs in Practical Application” was held in May 2017. Below, we report on the results of this meeting as well as on the background of the project and its goals.

## 2. Project “International Medical Students”

The project was founded in May 2016 at a general assembly of the German Medical Students' Association (bvmd) by a group of medical students from different medical schools who were already supporting international medical students at their respective universities. Over time, the group grew and now consists of many different nationalities. The project has set out the following goals:

1. To offer nationwide networking opportunities and support to local initiatives
2. To sensitize medical schools and the public to the topics of internationality and diversity of the medical student community
3. To represent the interests of international medical students within the medical student community nationally

After an initial stage of self-organization, the focus is now set on nationwide networking among local support programs which are distributed throughout Germany and are individually adapted to the local structures. Workshops are planned to provide a space for such an exchange on opportunities, hurdles and best-practice examples. The symposium “International Medical Students – Support Programs in Practical Application” was the first event of the “International Medical Students” project to achieve the desired goals.

## 3. Planning the symposium

The symposium “International Medical Students – Support Programs in Practical Application” took place from the 12^th^ to 14^th^ of May 2017 at the Hannover Medical School (MHH) and was jointly organized by the project “International Medical Students” and the local initiative "Integration and Linguistic Start into Medicine at MHH"(IsiEMHH). Prior to this, a search for existing support programs for international medical students was carried out in order to identify possible symposium participants and to develop interesting topics for workshops. The search was carried out through an internet search engine and the databases Pubmed/Medline and DIMDI. In total, 33 local support programs were determined. 

The identified support programs for international medical students are characterized by a high degree of heterogeneity, as two studies show [12], [23]: A significant difference could be found in the composition of the initiatives, as their members are recruited to varying degrees from the student body and the faculty of medical schools. The methods used to support international medical students in their studies also vary between locations. Linguistic and subject-related tutorials – which mostly take place before the beginning of medical school or during the first semesters – form a substantial part of the offers. Furthermore, general student counseling with informational events at the start of medical school as well as buddy programs, in which couples of international and German students support each other, were found. Cultural events which often focus on the facilitation of contacts between students were also identified. Lastly, aspects of financial support and representation of interests were found less frequently.

### 3.1 Goals of the symposium

As the expected participants of the symposium came from different support programs with diverse local structures, the overarching topic of the symposium was the discussion about components of, problems with and solutions for support projects. The symposium was intended to facilitate a first exchange between dedicated students and faculty members and summarize common perceptions and ideas in order to aid the creation of future support programs.

#### 3.2 Participants of the symposium

As part of the search mentioned above, various German support projects were identified and invited to the symposium. In addition, the local medical student councils at German universities and the national associations of medical students in Germany, Austria and Switzerland were contacted. Faculty members were addressed through the email distributors of the German Society for Medical Education (GMA) and also via invitations to all study deaneries in Germany.

**Figure 1:**
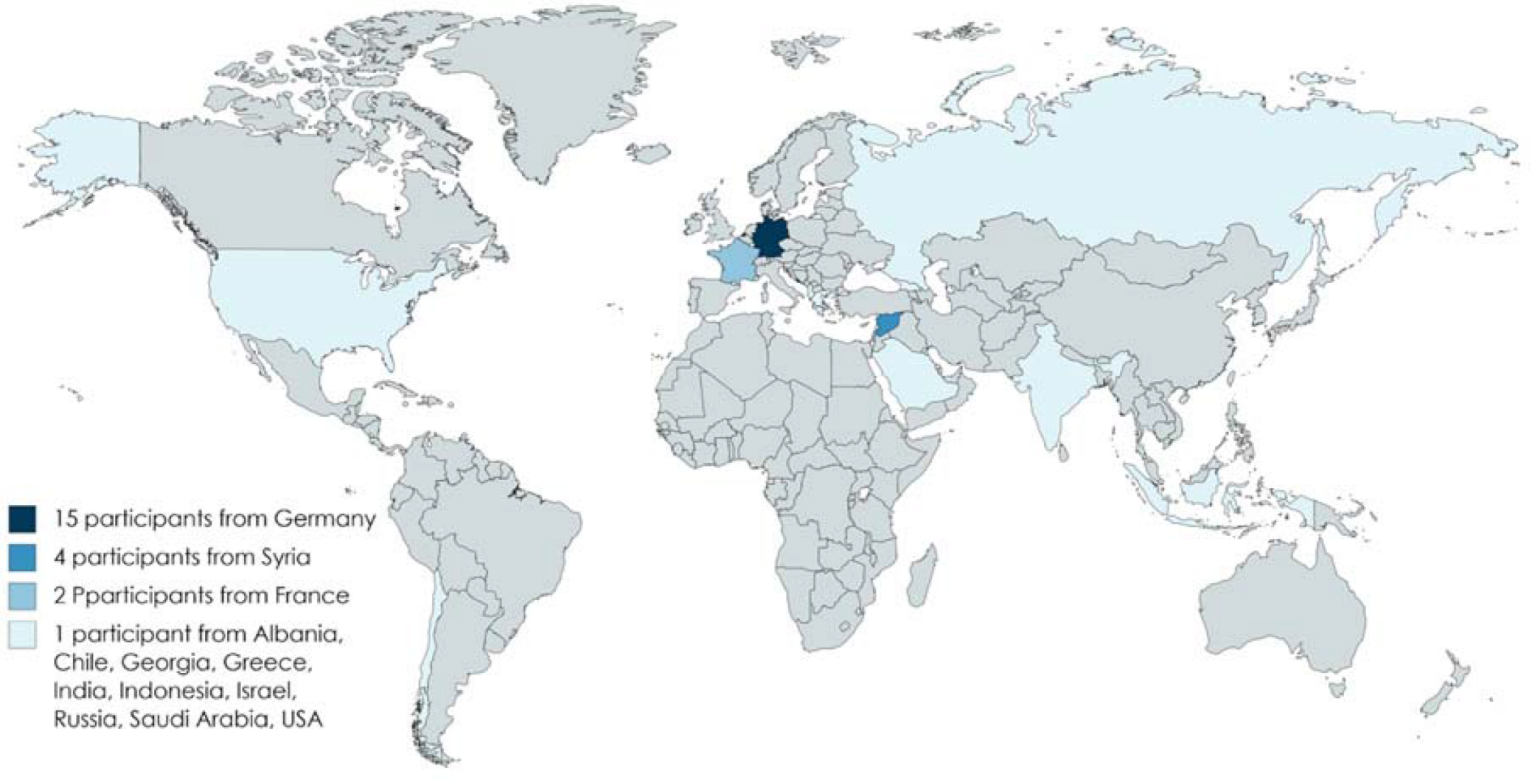
Countries of origin of the participants

31 participants from 10 German universities followed our invitation. They represented a large proportion of the previously known local initiatives as well as research groups. Although most participants were from Germany, 13 different nationalities were present at the symposium. Table 1 (Tab. 1) and Figure 1 (Fig. 1) summarize the characteristics of the workshop participants and the initiatives that were represented.

#### 3.3 Structure and schedule of the symposium

**Figure 2:**
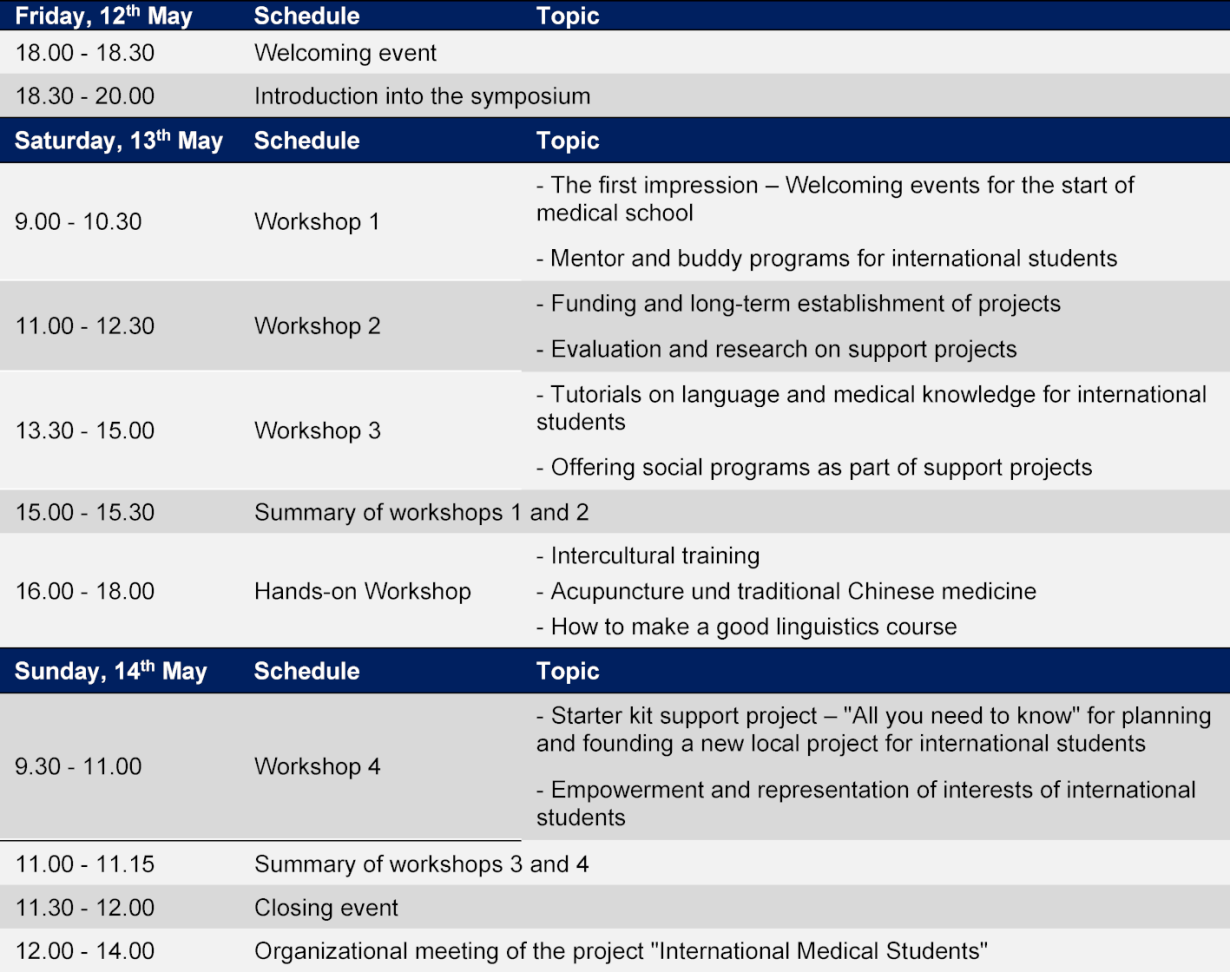
Schedule of the symposium “International Medical Students – Support Programs in Practical Application”

After an opening event, five workshop blocks were conducted over the weekend. Their respective topics are summarized in Figure 2 (Fig. 2). These workshops included a presentation of existing initiatives, their offers for international medical students and current problems in their local work. On this basis, the participants’ discussion and search for possible solutions took place.

## 4. Results

The results of the discussions can be summarized in four thematic blocks: general program structure, welcoming events, academic support and integration support.

### 4.1 General structures of a support program

The foundation stories of the local initiatives involved in the symposium were similar. Oftentimes, the motive for their foundation was the awareness of faculty members and students for the problems of international medical students. This, in turn, also poses a core problem to many small initiatives whose work is often based on the strong individual commitment of their members. A shortfall of these members subsequently weakens such initiatives. Thus the relevance of recruiting members was emphasized several times. In addition, a solid concept for the goals of the support program should be in place in order to remain able to act in the long term.

Project funding also presents a significant obstacle to many initiatives. The financial framework conditions at the various medical schools and medical student communities were heterogeneous and partly influenced by the respective state policies [https://studierendenvertretung-bayern.de/?page_id=100]. Although some initiatives were able to acquire third-party funding in the past (including funds from the German academic exchange service (DAAD) or the Ministry of Education (BMBF)), they were often limited by their funding periods. Thus, the early planning of financial needs plays a key role for local support programs, which is even more difficult for initiatives with many student members. Taking the required long-term continuity of initiatives into account, it was considered that essential funds for such projects have to be provided by the medical schools themselves. They can be supplemented by locally raised student funding, as these are less earmarked and more flexible in the use (i.e. for organizing cultural events).

Evaluations of events and initiatives were considered very important in the discussions, even though there was no indication that such evaluations were used by the represented support programs in a consistent way. Furthermore the methodological hurdles of such evaluations became evident. In particular, the typically small numbers of international medical students, their linguistic and cultural heterogeneity and strict data privacy policies were seen as potential obstacles. Especially smaller and purely student-organized initiatives can learn from the experience and methods of established initiatives as well as research groups in medical education research.

**Figure 3:**
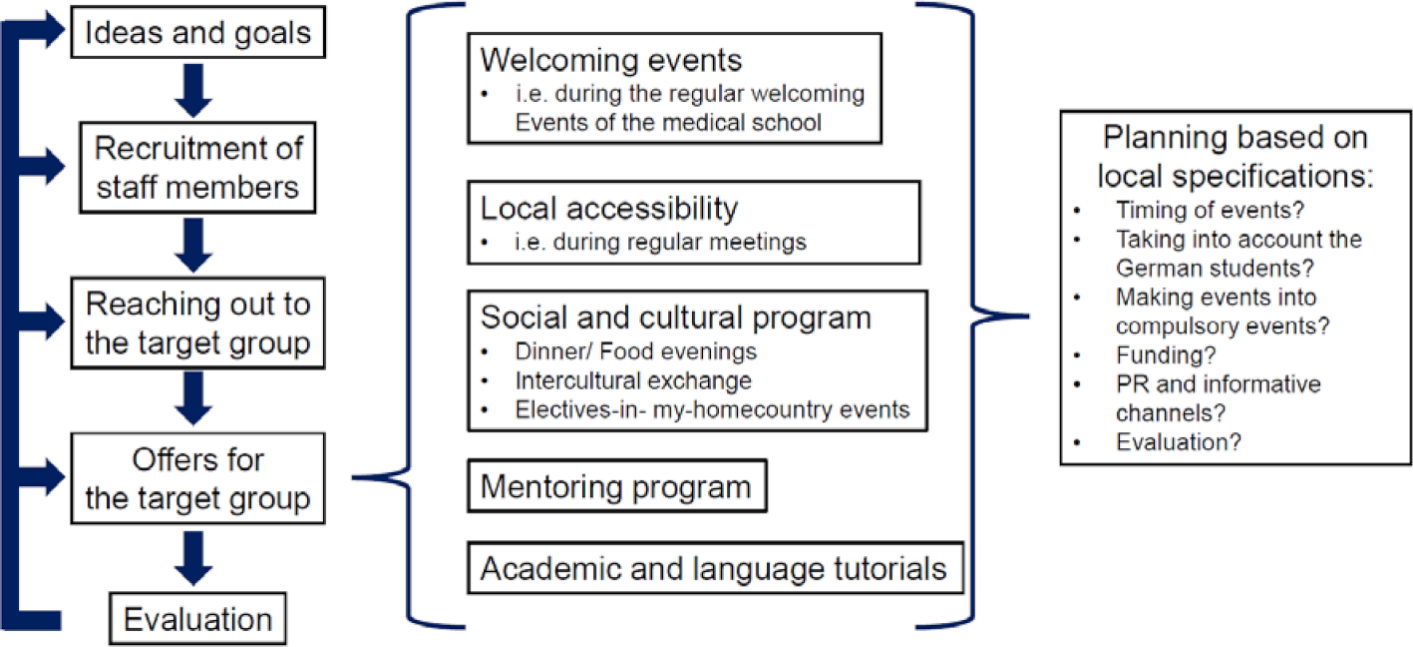
Possible steps in the development of a support program for international medical students

Figure 3 (Fig. 3) summarizes a selection of possible steps towards a local support program, as they have been discussed during the symposium. Some of the components of such a local support program are presented in the following sections of this article. 

#### 4.2 Welcoming events

**Figure 4:**
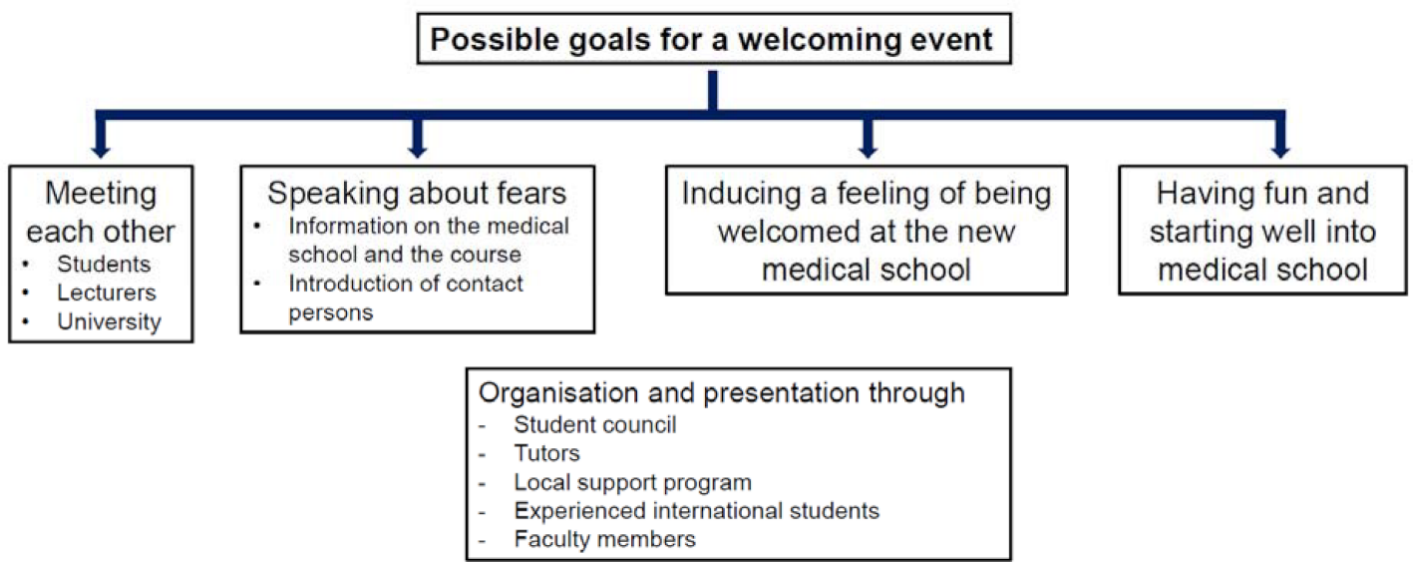
Possible goals for a welcoming event

Reaching out early to international medical students as well as providing them quickly with information and support are important factors that aid the success of international students. The support programs too would benefit from such early contact with the assisted international students. The work of local initiatives is strongly influenced by the start of medical school, since the actual recruitment of international medical students for the local support programs takes place here. The first impression that international students receive from a local support program has a large effect on a possible adherence to the program. This explains the widespread use of information and welcoming events for the start of medical school as well as subsequent events during the semester. These events often establish the necessary trust towards the initiatives that later enables struggling students to report their problems or experiences without fears. Welcoming events, which are often held by experienced students, also provide a platform for an informal exchange. They can reach out further than the official university counseling and are usually characterized by pragmatic approaches towards counseling. This, in addition to their high accessibility, turns them into a meaningful supplement to official offers by the university. Important goals of welcoming events are summarized in Figure 4 (Fig. 4).

Many initiatives perceive the start of medical school as a hurdle due to their competition with other groups for the attention of their targeted audience. This illustrates the relevance of a detailed analysis of the context and audience while planning the offers for international students. It is furthermore important that all events of a support project are well integrated into the students' weekly shedules and exam periods. In addition, a strong focus on public relations is helpful to reach both the targeted group of international students as well as possible new staff members for the support programs. To achieve this, the use of various channels (including social media, direct contact at welcoming events or information leaflets) is helpful. Addressing international students before their start into medical school is considered particularly promising, although it is often difficult for reasons of data privacy. However, prior consultation with local study deaneries and enrollment offices can often eliminate such concerns in advance.

The compulsory participation of international students in support programs was discussed controversially among workshop participants, as it was feared that participating students could experience disadvantages. Possible alternatives for such an approach were seen in the use of incentives (e.g. course certificates) for participating students. Furthermore it has to be noted that such incentives need to be attractive for international medical students to avoid low participation rates.

#### 4.3 Academic support

Academic support for international medical students is a key element of many local initiatives. It is often implemented in form of linguistic [25], [26] and subject-related tutorials [16], [17], [18] as well as mentoring programs [16], [18]. The expectation of specific support in coping with academic challenges makes such offers particularly interesting for international medical students. Also, tutorials are often the longest-standing components of initiatives, providing the organizing programs with a large amount of expertise in the design and implementation of such offers. 

The discussion about academic tutorials showed that these are often taught by peers from higher semesters and evaluated positively by the participants. On the one hand, the slower linguistic repetition of the academic subjects was emphasized. This enables the accentuation of aspects with relevance to later examinations, as they could not always be filtered out by the international students in the regular class. This illustrates that the application of the German language does indeed challenge many international medical students. Therefore, tutorials are occasionally offered with a focus on specific linguistic support (i.e. through lecturers for German as second language). On the other hand, the tutorials at medical schools have different ways of communicating their content and the discussion showed that the academic and linguistic elements of tutorials cannot be considered separately. This becomes even more evident over the course of medical school when medical terminology has to be applied in different communication contexts (i.e. patient interviews). The question for the optimal structure of tutorials in the medical curriculum could not be sufficiently answered due to the many differences among the represented local support programs. Nevertheless, it became clear that an active participation of international medical students can be considered beneficial for later oral examinations.

Mentoring programs were seen as another element of academic support. Based on their own experience, students from higher semesters should ideally serve as mentors. In addition, students with a similar linguistic, social or cultural background could build good relationships with their mentees through social and cognitive congruity. However, according to some participants of the symposium, the voluntariness of mentoring programs can be challenging for the organization (i.e. the recruitment of new mentors) of such programs. Here too, incentives for mentors and mentees can be supportive.

#### 4.4 Promoting integration

Offers for the promotion of integration of international medical students are relatively young in comparison to academic and linguistic support offers. They are often initialized and carried out by committed student members of support programs. The discussion on measures to promote integration underlined the importance of the context of such offers. Neutral meeting rooms were considered well-suited to enable international students to easily contact other students and build friendships. Additionally, cultural evenings, dinner events or excursions [16], [18] were seen as helpful tools to emphasize the diversity and strengths of an international student community. The participation of German students in the offers of support programs was discussed extensively in the context of integration efforts. An advantage was seen in the prevention of group formation among international students and the promotion of integration into the larger student community. It was assumed that this could support learning between students with different cultural and linguistic backgrounds. Cultural mixing with German students was only seen as a possible disadvantage in the case of academic tutorials where the desired framework conditions (i.e. the focus on linguistic aspects in the subject as well as a fearless talk about comprehension problems) appeared vulnerable to changes in group composition. Nevertheless, this could reinforce a perception of the low academic performance of international medical students at the same time.

The empowerment and advocacy of international medical students was discussed as another aspect of integration. As well as through strengthening the visibility of diversity within the student body, this could contribute to a reduction in deficit orientation towards international medical students. Possible examples for such empowerment were seen in events such as the International Day (Heidelberg) or country-related film evenings followed by discussions (MHH), in which international students contribute their perspectives. Nevertheless, it must be remembered that the organization of such events requires a large amount of time which only few international students can provide under the current conditions.

## 5. Evaluation of the symposium

16 individuals participated in the online evaluation of the symposium. In addition to the assessment of the actual planning of the symposium, the question of the perceived benefits of the exchange between local initiatives was evaluated. It turned out that the topics of the symposium seemed to suit most of the participants, although the timing was not always perceived as sufficient. In particular, some participants would have wished to have more time for the personal exchange between local initiatives. Also, there was an interest in further discussions on academic and linguistic tutorials. In turn, the aspect of empowerment of students received a comparatively low interest in the evaluation results.

## 6. Conclusions and perspectives

The present report from the first national symposium “International Medical Students – Support Programs in Practical Application” represents a first step towards a regular exchange between local support programs for this group of students. Due to the increasing number of international medical students [1] and the rising number of foreign physicians in German hospitals [27], it is important to address the specific intercultural challenges in medical education and training. During the symposium these as well as possible solutions were mentioned and discussed. It appeared that many initiatives are already pursuing similar principles in supporting their international students. In particular, the promotion of academic and linguistic competencies as well as the integration into the local student body play a central role here. At the same time, the framework conditions found at the individual medical schools, in the general field of medical education and in the funding of local support projects present a number of challenges that inhibit local work. Solutions to these challenges are not yet widely established and local circumstances often have to be taken into consideration. 

In particular, the question of what is the most successful way of supporting international medical students can only be answered inadequately. A variety of support approaches could be seen during the symposium, but their impact has rarely been evaluated so far. To do this, medical schools not only need to establish further support programs that design events for international medical students, but also increase their research efforts to identify promising approaches among these programs.

The same applies to the question of the long-term goals of support projects. Currently there is a focus on study problems of international medical students. Although this is pragmatic in the context of achievement orientation in medical education, it subsequently leads to a deficit orientation towards international medical students [28]. Nevertheless, these students do not only retain teaching resources, but also offer great potential to their medical schools [29]. An international campus promotes cross-cultural communication, integration and thus an intercultural learning atmosphere. In the long term, this can lead to worldwide cooperation. As part of their education, students can be prepared in optimal ways for the cultural diversity of the health system and medical schools can use their cultural diversity of their student bodies to attract more international students.

To implement the ideas mentioned in this article, a successful collaboration between medical schools and committed students is required. In addition, a common strategy on how to better support and promote international medical students in the future could raise awareness to this important topic and provide new funding opportunities for support programs and constructive discussions.

The positive feedback on the need for regular networking meetings between local initiatives is a core message from this year's symposium by the organizing project “International Medical Students”. Due to open questions on the evaluation of offers for international medical students as well as the desire for qualification and training for local initiatives, a following symposium will be organized from 01.-03. June 2018 in Heidelberg.

## Funding

The symposium was funded through participation fees as well as official sponsoring by the MHH plus Foundation and the MHH Alumni Association.

## Acknowledgements

The authors would like to thank all participants of the symposium for the exciting and stimulating discussions. Furthermore, we would like to thank the supporters of the conference. These include the MHH plus Foundation and MHH-Alumni Association as well as the members of the IsiEMHH project who have taken over the organization on site. 

## Competing interests

The authors declare that they have no competing interests. 

## Figures and Tables

**Table 1:**
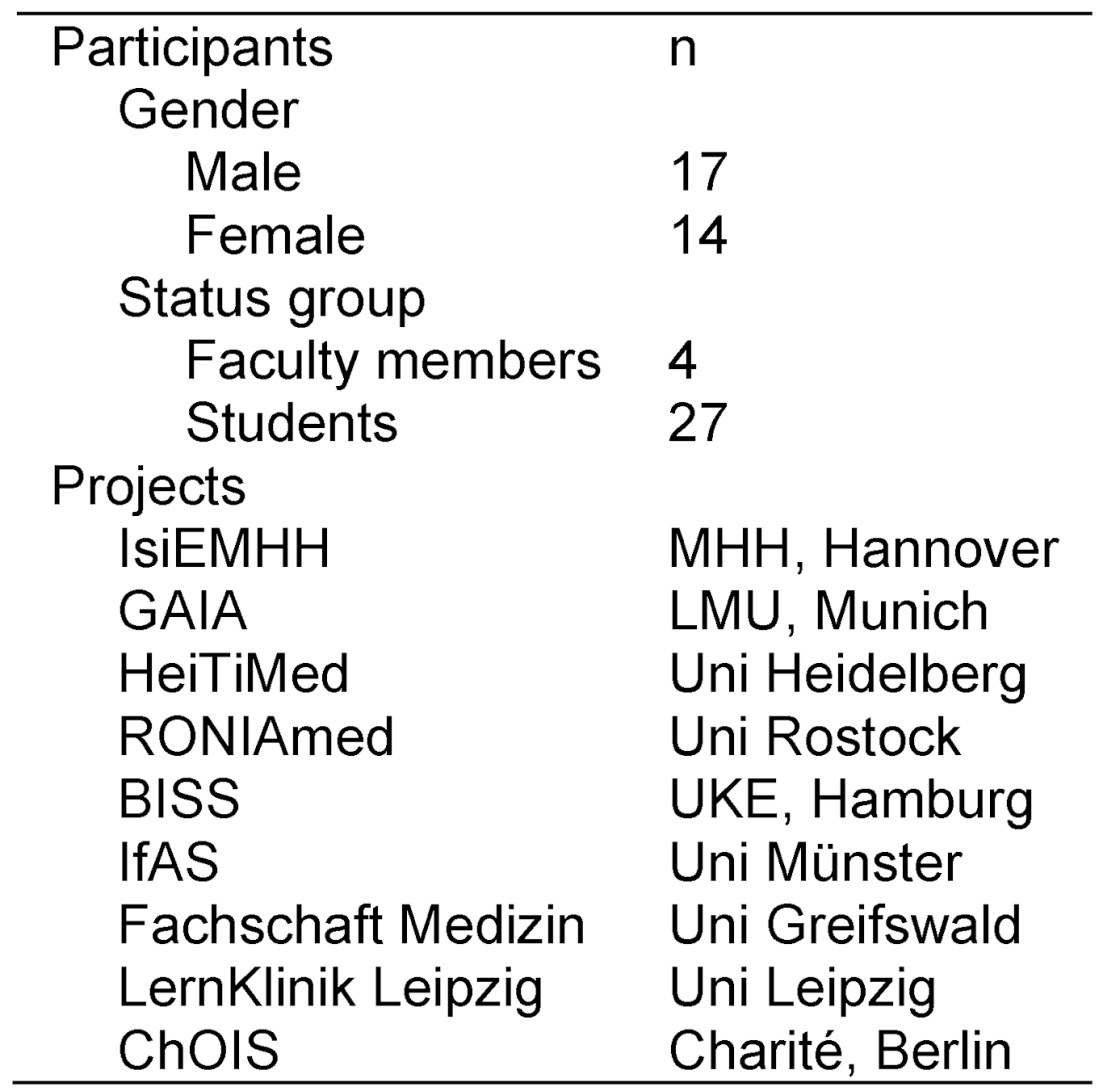
Participants and their local projects
